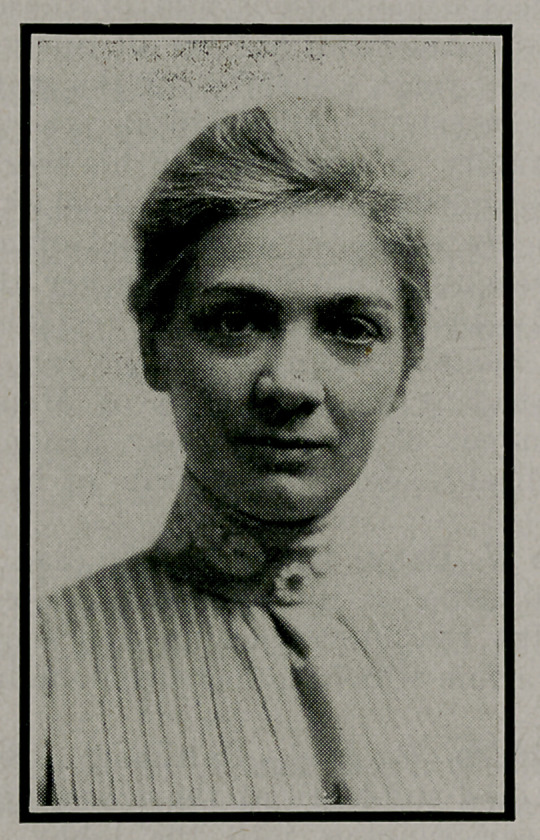# In Memoriam: Dr. Ida C. Bender

**Published:** 1916-08

**Authors:** Nathalie Mankell, Jeanette Hummelsbach, Marie Wolcott, Alice Bennett

**Affiliations:** President; Committee; Committee; Committee


### IN MEMORIAM

Whereas, It has pleased God in his mercy to take the spirit of our beloved member into His kingdom, we the Physicians'

League feel that our esteemed friend, Dr. Ida C. Bender, always stood for the highest and best in the field of her activity, education and medicine, and applied the very best the world has to give to the mental uplift and the physical well being of the youth of this community,

Whereas, She held a position of the first rank in the educational world and applied her medical knowledge in a broader and more far-reaching way than the average physician has the opportunity of doing,

Whereas, We always appreciated Dr. Bender's rare personality which exerted a beneficent influence on all who came within the sphere of her influence, to higher and better things,

Therefore, Be it resolved that the Physicians' League extend to the family and Miss Louise M. Lapey, her friend of lifelong devotion, our deepest sympathy in their bereavement, and that we cause to be sent to the family a copy of these resolutions.

DR. NATHALIE MANKELL,
President.
DR. JEANETTE HUMMELSBACH,
DR. MARIE WOLCOTT,
DR. ALICE BENNETT,
**Committee.**

## Figures and Tables

**Figure f1:**